# Effects of Moisture Content Gradient on Alfalfa Silage Quality, Odor, and Bacterial Community Revealed by Electronic Nose and GC–MS

**DOI:** 10.3390/microorganisms13020381

**Published:** 2025-02-09

**Authors:** Yichao Liu, Zhijun Wang, Lin Sun, Yuhan Zhang, Muqier Zhao, Junfeng Hao, Mingjian Liu, Gentu Ge, Yushan Jia, Shuai Du

**Affiliations:** 1 Key Laboratory of Forage Cultivation, Processing and High Efficient Utilization, College of Grassland Science, Inner Mongolia Agricultural University, Hohhot 010010, China; 2 Inner Mongolia Academy of Agricultural and Animal Husbandry Sciences, Hohhot 010010, China; 3 Forestry and Grassland Work Station of Inner Mongolia, Hohhot 010000, China

**Keywords:** fermentation quality, odor composition, volatile organic compounds, bacterial diversity, metabolic pathway

## Abstract

Better quality and odor of silage and normal microbial fermentation metabolism are mostly dependent on an appropriate moisture content. The purpose of this study was to determine the effects of different moisture content gradients (50, 60, 70, and 80%) on the bacterial community, odor, and quality of alfalfa silage at 60 days by using gas chromatography–mass spectrometry (GC–MS) and electronic nose, with six replicates per group. The results showed that there were significant differences in odor response intensity among all groups, among which the 80% group had the strongest reaction to terpenoids, sulfides, and nitrogen oxides. Similarly, the different volatile organic compounds (VOCs) were mainly terpenoids, alcohols, and ketones, such as pine, camphor, and menthol (e.g., carlin and levomenthol). The dominant bacterium was *Enterococcus* with higher fiber, pH, and ammonia nitrogen (NH_3_-N) content but poorer quality and odor (*p* < 0.05). The differential VOCs in the 60% group were mainly heterocyclics, esters, and phenols with fruity, floral, and sweet odors such as 2-butylthiophene and acorone. *Pediococcus* and *Lactiplantibacillus* were the dominant bacteria, with higher crude protein (CP), water-soluble carbohydrates (WSC), and lactic acid (LA) contents, as well as better quality and odor (*p* < 0.05). The biosynthesis of terpenoids and steroids, biosynthesis of secondary metabolites, and biosynthesis of phenylpropanoids were the main metabolic pathways of differential VOCs. In conclusion, regulating moisture content can alter bacterial community and metabolites, which will encourage fermentation and enhance alfalfa silage quality and odor.

## 1. Introduction

Silage is a method of fermenting and preserving feed under anaerobic conditions. The success of silage fermentation directly affects the nutritional value and storage time of the feed, and the moisture content plays a crucial role in silage fermentation [1]. The moisture content refers to the proportion of water contained in the silage, and the appropriate moisture content helps the silage to be compacted, to exclude air, and to form a good anaerobic environment. This is crucial for silage fermentation because the anaerobic environment inhibits the growth of aerobic microorganisms such as molds and yeasts and promotes the reproduction of anaerobic microorganisms such as lactic acid bacteria [2]. The moisture content of alfalfa silage is usually 60% to 70%, and the fermentation effect is good. Excessive moisture content will cause the feed to be difficult to compact, and the air will be difficult to remove, which is not conducive to the formation of an anaerobic environment and will also lead to nutrient loss and increase the risk of spoilage [3]. When the moisture content is too low, it will make the feed difficult to compact, and the residual air will increase, resulting in incomplete fermentation and high nutrient loss.

The process of silage fermentation includes the initial aerobic stage, the anaerobic fermentation stage, and the stable stage, and the water content has an important influence on each stage [4]. During the aerobic stage, the appropriate moisture content can promote the rapid compaction of silage, reduce the residual oxygen, shorten the aerobic stage, and prevent excessive nutrient loss [5]. In the anaerobic fermentation stage, lactic acid bacteria ferment the sugars in the feed into lactic acid, and the appropriate moisture content helps the activity of lactic acid bacteria to ensure smooth fermentation, rapidly reduce the pH value, and inhibit the growth of harmful microorganisms [6]. When the pH of silage is reduced to a stable level, the fermentation process tends to stabilize, and the appropriate moisture content at this time helps to maintain the acidic environment of the silage and prevent secondary fermentation and spoilage [7].

Previous studies have shown that the moisture content of silage directly affects its nutrient composition and preservation effect. At the same time, the moisture content will also have a significant effect on the odor of silage fermentation. The odor is an important sensory index to measure the quality of silage, and a bad odor will not only affect the palatability of livestock but may also indicate that there are problems with the fermentation of the feed [8]. At the appropriate moisture content, lactic acid is produced by lactic acid bacteria fermenting in an anaerobic environment, causing the silage to give off a fresh sour smell, indicating a smooth fermentation process and good silage quality. When fermentation is abnormal, a foul or pungent odor is produced, and silage quality deteriorates severely [9]. Electronic nose (E-nose) and gas chromatography–mass spectrometry (GC–MS) are two important techniques used for odor detection and analysis in modern analytical chemistry. E-nose can be used as a rapid screening tool to detect the odor characteristics of samples and determine whether further analysis is needed [10]. For the screened differential samples, detailed composition analysis using GC–MS can accurately quantify and identify volatile organic compounds. The electronic nose can provide comprehensive odor characteristic information of silage through pattern recognition technology, which is suitable for rapid evaluation, but it cannot determine the composition and quantification of specific compounds. GC–MS enables accurate qualitative and quantitative analysis of volatile compounds in silage, providing detailed composition information [11]. Stevan et al. [12] showed that the E-nose was able to distinguish between different varieties and seasons of fermented blueberry wine. Zhou et al. [13] used an E-nose combined with GC–MS to reveal the trend of black tea fermentation over time and divided the fermentation process into three stages. In recent years, E-nose and GC–MS have been used with satisfactory results for the quality, fermentation stage, and origin of fermented foods. However, few studies have been carried out on the combination of electronic nose and gas chromatography for the analysis of silage odors.

Alfalfa is a forage with a high moisture content (80–90%), and the selection of appropriate moisture for silage may have an impact on its ensiling effect and microbial community. Current research on moisture content mainly focuses on silage quality, while less research has been conducted on its effect on silage odor. The aim of this study was to use an E-nose combined with GC–MS to reveal the effects of moisture content gradient on alfalfa silage quality, bacterial composition, and volatile organic compounds and to determine the appropriate moisture content to improve silage quality and odor.

## 2. Materials and Methods

### 2.1. Silage Preparation

The variety of alfalfa raw material was WL319HQ, planted in Tuerte Left Banner, Hohhot City, Inner Mongolia, and the harvest time was the first flowering period (25 June 2023). By controlling the drying time, four gradients of the moisture content of the raw material under natural drying conditions were set: 50%, 60%, 70%, and 80%. After cutting the forage into a length of 2–3 cm, 400 g of it was vacuum-sealed in polyethylene bags (25 × 30 cm) and stored at room temperature, with 6 bags for each treatment. After silage for 60 days, silage quality, microbial community, and volatile organic compounds were evaluated.

### 2.2. Response Intensity Analysis

The odor response intensity of the samples was measured using a PEN3 electronic nose from Ensoul Technology Ltd., Schwerin, Germany [14]. The performance characteristics of each sensor in the electronic nose can be found in Table 1. A 2 g sample was placed into a 20 mL sample bottle, and the injection needle was cleaned for 95 s before sampling. The zero point was adjusted for 5 s, and the preparation time was 5 s. The initial injection flow rate and the sample chamber flow rate were both set to 300 mL/min [15]. The measurement time was 100 s, with data recorded every 2 s during an acquisition interval of 1 s. Furthermore, prior to the subsequent injection, the system required a 95 s period to cleanse the injection needle in order to establish a stable baseline. The odor response intensity was determined by calculating the ratio of the resistance of the volatile gases in the sample (G) to the resistance of the corresponding carrier gas (G0) over time [16].$\;Response\;intensity=\frac{G}{G0}$.

### 2.3. Silage Quality Analysis

The samples underwent a drying procedure for a duration of 72 h at a temperature of 65 °C in order to ascertain their dry matter content (DM). The crude protein (CP) was assessed using the Dumas nitrogen determination technique employing a Dumas-01 model manufactured by Gerhardt Analytical Instruments Co., Ltd., Königswinte, Germany [17]. An ANKOM fiber analyzer (Model: A2000i), manufactured by Beijing ANKOM Technology Co., Ltd., Beijing, China, was used to measure the amounts of acid detergent fiber (ADF), and neutral detergent fiber (NDF) [18]. An ANKOM fat analyzer (Model: XT15i) from Beijing Anke Borui Technology Co., Ltd., Beijing, China, was used to analyze crude fat (EE). The quantification of water-soluble carbohydrates (WSCs) was performed using anthrone colorimetry [19]. The pH level was measured using an acidity meter from Shanghai Yida Scientific Instruments Co., Ltd., Shanghai, China (Model: LEICI pH S-3C). Lactic acid (LA), acetic acid (AA), propionic acid (PA), and butyric acid (BA) were identified using high-performance liquid chromatography with a Waters e2695 model from Massachusetts, MA, USA [20]. In addition, the concentration of ammonia nitrogen (NH_3_-N) was measured using the phenol–hypochlorite colorimetric method [21].

### 2.4. Bacterial Diversity Analysis

The genomic DNA was extracted using the cetyltrimethylammonium bromide (CTAB) method, and its purity and concentration were assessed using agarose gel electrophoresis. The DNA samples were collected in centrifugal tubes and then diluted to a concentration of 1 ng/L using sterile water. The sequenced primers were 799F (AACMGGATTAGATACCCKG) and 1193 R (ACGTCACCCCTTCC). PCR was performed to amplify the V5–V7 region of bacterial 16S rRNA using Phusion High-Fidelity PCR Master Mix and GC Buffer from New England Biolabs, along with efficient and high-fidelity enzymes. The libraries were generated utilizing the TruSeq DNA PCR, a complimentary sample preparation kit, and subsequently sequenced on the NovaSeq6000 platform. Barcode sequences and PCR-generated primer sequences were utilized for downstream data analysis. The extraction of pertinent labels was performed using FLASH (v1.2.11) and QIIME (v1.9.1) on the samples. The tags were subsequently grouped together into Operational Taxonomic Units (OTUs, with a threshold of 97%) using USEARCH software version 7. Species annotations were conducted on sample sequences of each Operational Taxonomic Unit (OTU) using SILVA (version 138.1). The abundance and diversity within QIIME were measured using MAFFT software (version 7.490) for efficient comparison of several sequences. PICRUSt2 was utilized to forecast and annotate microbial function based on 16S rDNA data and the Kyoto Encyclopedia of Genes and Genomes (KEGG).

### 2.5. Volatile Organic Compound Analysis

The samples were collected, immediately preserved in liquid nitrogen, and thereafter stored at a temperature of –80 °C. The sample was compressed using liquid nitrogen, and 500 mg of the resulting powder was combined in 20 mL vials. To avoid enzyme reactions, a concentrated solution of sodium chloride (NaCl) was added, and the vial was tightly sealed with a flexible silicone cap. Prior to solid-phase microextraction, each vial was subjected to heating at a temperature of 60 °C for a duration of 5 min. An Agilent fiber optic was thereafter introduced into the headspace vials and maintained at a temperature of 60 °C for a duration of 15 min in order to collect volatile organic chemicals present in the sample. An Agilent 8890 7000D mass spectrometer was used to detect and measure volatile organic molecules. A helium-filled DB-5MS capillary column containing 5% phenyl-polymethylsiloxane was utilized to intake air at a flow rate of 1.2 mL/min. The temperature of the injector was held constant at 250 °C, while the temperature of the detector was adjusted to 280 °C. The initial oven temperature was set to 40 °C for a duration of 3.5 min, after which it rose at a rate of 10 °C per minute until it reached 100 degrees. Subsequently, there was an increase of 7 °C per minute, reaching a temperature of 180 °C. This was then followed by a further increase of 25 °C per minute, reaching a temperature of 280 °C. Finally, the temperature was maintained at this level for a duration of 5 min. The mass spectra were obtained using the electron impact (EI) ionization technique at an energy level of 70 electron volts (eV). The temperatures of the quadrupole mass detector, ion source, and transmission lines were 150 °C, 230 °C, and 280 °C, respectively. The chemical was identified and quantified using the selective ion monitoring (SIM) technique. The MassHunter quantitative program was utilized to conduct integration and calibration correction processes, with 3-hexanone (CAS 24588–54–3) serving as the internal standard [22].

### 2.6. Statistical Analysis

This study utilized SAS 9.2 to conduct an analysis of variance (ANOVA), with six replicates in each group. Statistical significance was assessed based on *p*-values that were lower than 0.05. The dependability of the sample means was evaluated using the standard error of the mean (SEM). The orthogonal partial least square method (OPLS-DA) was used to analyze the sample differences, and the OPLS-DA evaluation model was used to verify the data for 200 random permutations and combinations. The tables were created using Microsoft Excel 2010, while the graphs were produced using GraphPad Prism 8.0.2 and R 4.1.2.

## 3. Results

### 3.1. Odor Response Intensity of Alfalfa Silage

**Figure 1:**
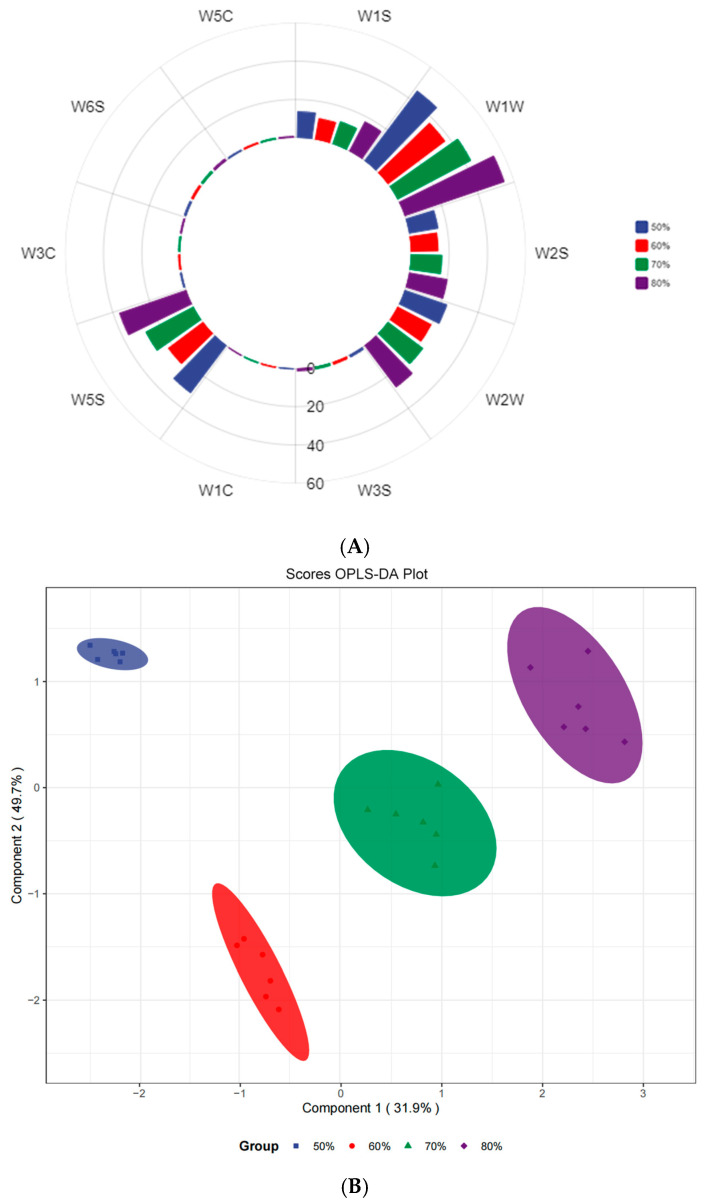

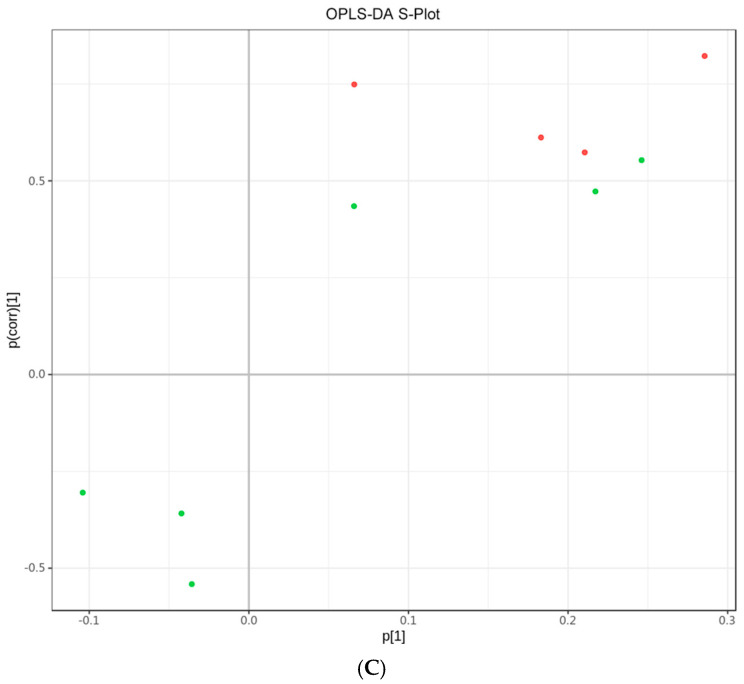
Radar plots of response intensity for each group (**A**). OPLS-DA score plot of response intensity (**B**). OPLS-DA S-plot of response intensity (**C**). Each point represents a substance (metabolite/protein/gene, etc.). The closer the two corners, the more important the variable; the red point indicates the VIP value of the substance >1, and the green point indicates the VIP value of the substance <1.

The electronic nose can distinguish the odor of the sample through the response intensity of the sensor. The odor characteristics of alfalfa silage at different moisture contents are shown in Figure 1A. The sensors with high response strength to different moisture contents of alfalfa silage were W1W, W5S, W2W, W1S, and W2S, which corresponded to terpenes, inorganic sulfides, nitrogen oxides, organic sulfides, methyl groups, alcohols, aldehydes, and ketones. The most obvious differences among samples were W1W, W5S, and W2W, indicating that the moisture content had a great influence on the terpenes, inorganic sulfides, nitrogen oxides, and organic sulfides of alfalfa silage. Among them, the response intensity of these three sensors in the 80% group was the largest, followed by the 50% group and the 70% group, with the 60% group being the smallest.

Orthogonal partial least squares discriminant analysis (OPLS-DA) is able to show the differences between and within the sample groups, where the horizontal direction represents the differences between the groups, the vertical direction represents the differences within the groups, and the percentage represents the interpretation rate. Figure 1B shows that alfalfa silage samples with different moisture contents have obvious separation among groups and relatively large aggregation within groups, indicating that there are significant differences in odor among groups and small differences within groups (R2X = 0.916, R2Y = 0.985, and Q2 = 0.952; the model was fitted with high accuracy). Among them, the 50% group, 60% group, and 80% group were far distributed, indicating that the odor characteristics of low-moisture-content and high-moisture-content alfalfa silage were different. The results of the OPLS-DA S–plot of response intensity show that the odor substances corresponding to W1W, W2W, W1S, and W5S sensors contribute more to alfalfa silage.

### 3.2. Nutrition and Fermentation Quality of Alfalfa Silage

The nutritional quality of each group is shown in Table 2, and moisture content had significant effects on each index (*p* < 0.05). The DM content in each group was 50%, 60%, 70%, and 80%, in the order of highest to lowest. The CP content of the 60% group was significantly higher than that of other groups (*p* < 0.05), and the WSC content was significantly higher than that of the 50% and 80% groups (*p* < 0.05), with no significant difference compared to the 70% group. Its NDF content was significantly lower than that of the other groups (*p* < 0.05), and its ADF content was significantly lower than that of the 50% and 80% groups (*p* < 0.05), with no significant difference compared to the 70% group. The content of EE was significantly higher than that of the 50% and 80% groups (*p* < 0.05), with no significant difference compared to the 70% group.

The fermentation quality of each group is shown in Table 3, and moisture content had significant effects on each index (*p* < 0.05). The pH and NH_3_-N contents of the 60% and 70% groups were significantly lower than those of the 50% and 80% groups, while the pH and NH_3_-N contents of the 80% group were significantly higher than those of other groups (*p* < 0.05). The content of LA in the 60% and 70% groups was significantly higher than that in the 50% and 80% groups, and the content of LA in the 80% group was significantly lower than that in the other groups (*p* < 0.05). The AA content of the 60% group was significantly higher than that of the other groups, and the AA content of the 80% group was significantly lower than that of the other groups (*p* < 0.05).

### 3.3. Bacterial Composition of Alfalfa Silage

The α-diversity of each group is shown in Table 4. The Otus value of the 80% group was significantly higher than that of other groups, and the Otus value of the 50% and 70% groups was significantly lower than that of the 80% group (*p* < 0.05). The Shannon value of the 50% and 80% groups was significantly higher than that of the other groups, and the Simpson value of the 70% group was significantly lower than that of the other groups (*p* < 0.05). Chao1 and ACE in the 80% group were significantly higher than those in the other groups, and Chao1 and ACE in the 50% group were significantly lower than those in the other groups (*p* < 0.05).

**Figure 2:**
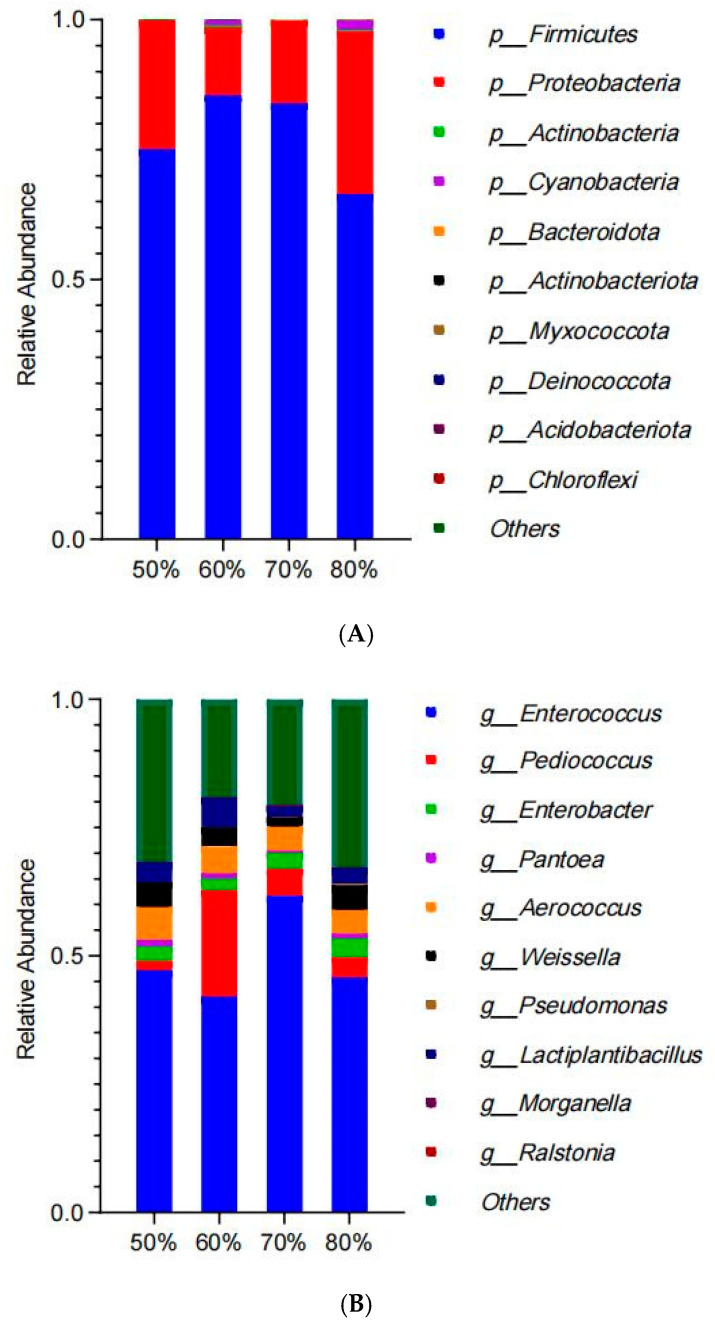

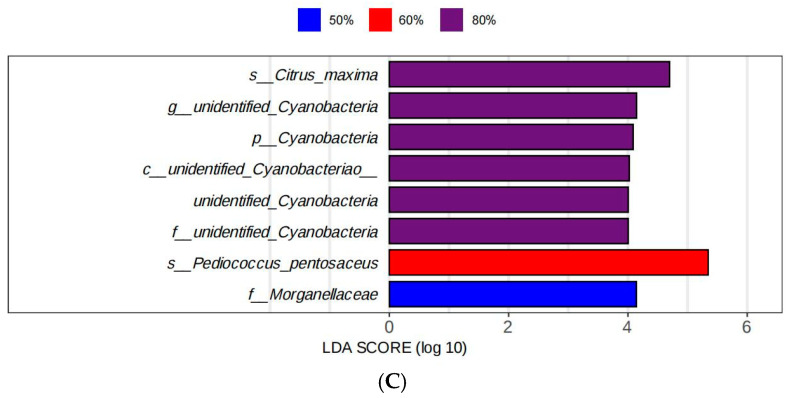
Bacterial composition of alfalfa silage at phylum (**A**) and genus (**B**) levels. LEfSe analysis of bacterial composition in alfalfa silage (**C**).

The composition and relative abundance of bacteria at the phylum level is shown in Figure 2A. The relative abundance of *Firmicutes* and *Proteobacteria* was higher in all groups, where the abundance of *Firmicutes* was highest in the 60% group, followed by the 70%, 50%, and 80% groups, in descending order. The relative abundance of *Proteobacteria* in the 50% and 80% groups was higher than those in other groups. The composition and relative abundance of bacteria at the genus level is shown in Figure 2B. The bacterial community was mainly composed of *Enterococcus*, *Pediococcus*, *Enterobacter*, *Aerococcus*, *Weissella*, and *Lactiplantibacillus*. Among them, the relative abundance of *Enterococcus* was higher in all groups, with the highest value in the 70% group, followed by the 50%, 80%, and 60% groups, in descending order. The relative abundance of *Pediococcus* in the 60% group was significantly higher than that of other groups, and the relative abundance of *Lactiplantibacillus* was also higher. The LEfSe analysis results for bacterial composition are shown in Figure 2C. *f_Morganellaceae* in the 50% group contributed significantly to the difference between groups. *s_Pediococcus_pentosaceus* in the 60% group contributed significantly to the difference between groups. *s_Citrus_maxima* and *p_Cyanobacteria* in the 80% group contributed significantly to the difference between groups.

### 3.4. Functional Prediction of Bacteria

**Figure 3:**
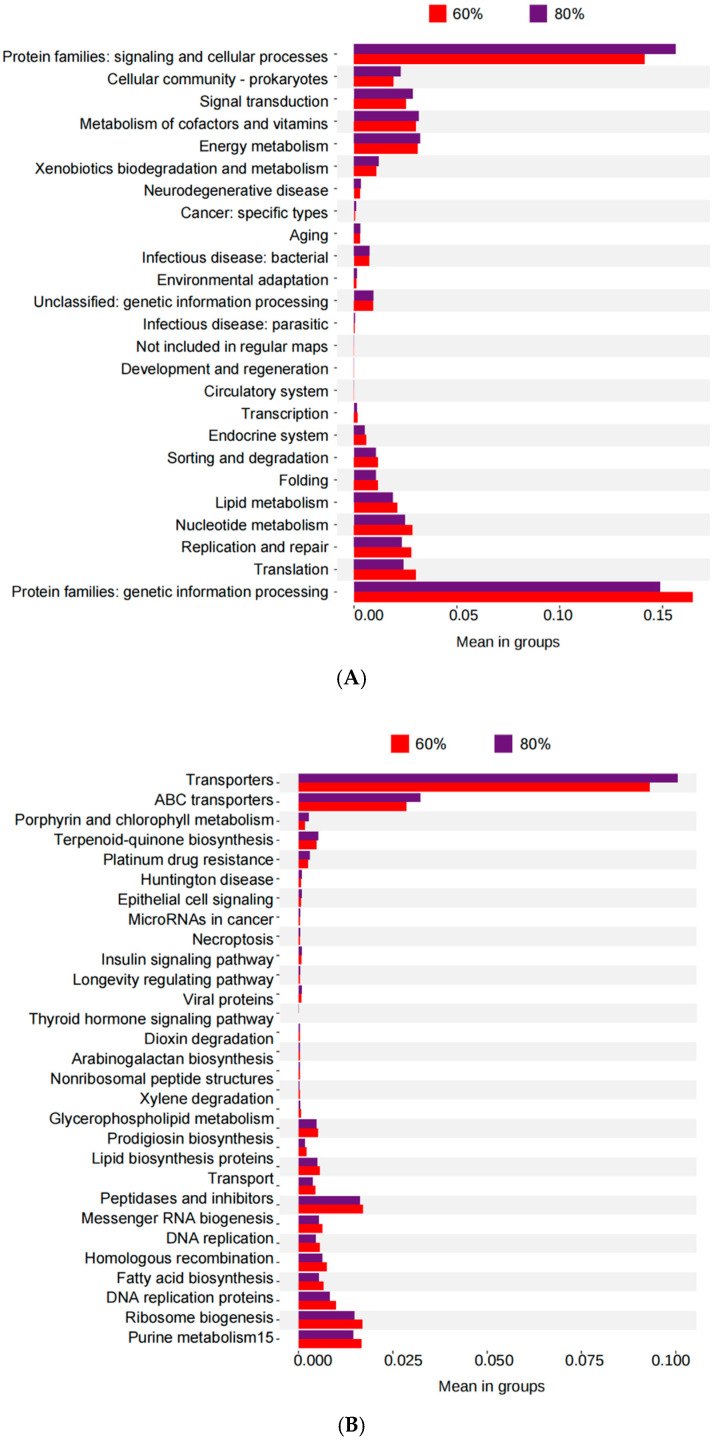
Microbial function prediction at level 2 (**A**) and level 3 (**B**).

The 60% and 80% groups with large differences in odor characteristics and quality were selected for functional prediction and comparison based on the KEGG database, and the results are shown in Figure 3. Among the main functions of the L2 level, the abundance of protein families regarding genetic information processing, translation, replication and repair, and nucleotide metabolism was higher in the 60% group than in the 80% group, but the abundance of protein families regarding signaling and cellular processes, energy metabolism, and the metabolism of cofactors and vitamins was higher in the 80% group. Among the main functions of the L3 level, the abundance of transporters, ABC transporters, terpenoid-quinone biosynthesis, porphyrin and chlorophyll metabolism, and platinum drug resistance was higher in the 60% group than in the 80% group. The peptidases and inhibitors, purine metabolism, ribosome biogenesis, DNA replication proteins, and fatty acid biosynthesis were less abundant than in the 80% group.

### 3.5. Differential VOCs in Alfalfa Silage

**Figure 4:**
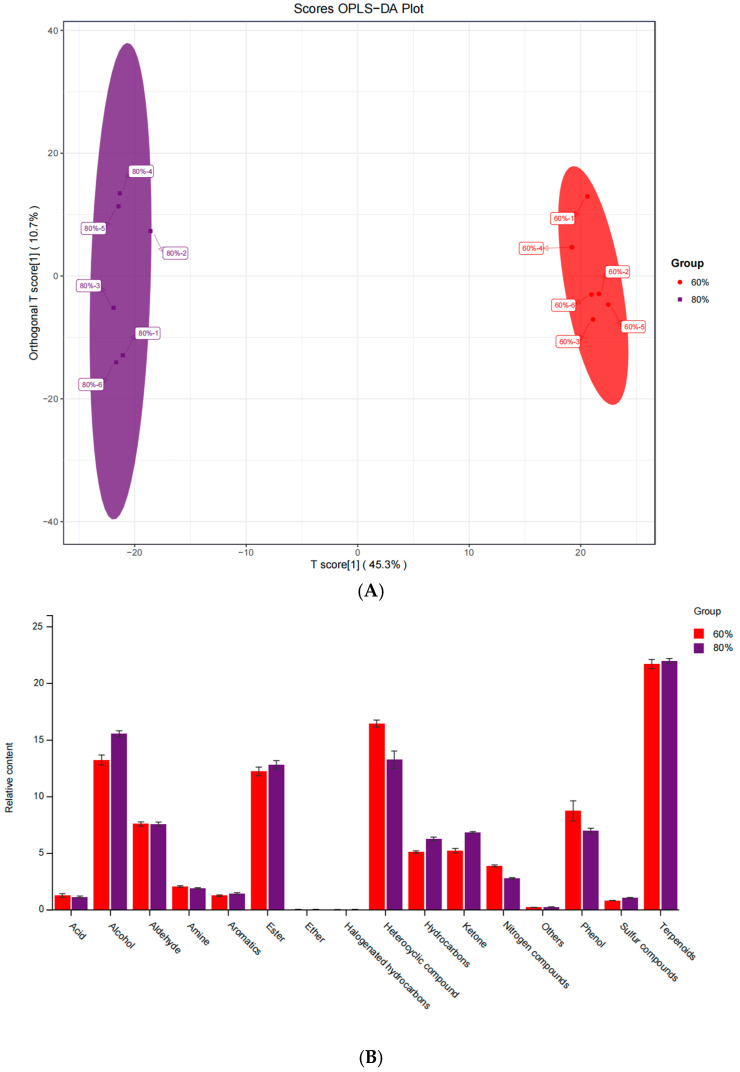
OPLS-DA score plot of VOCs (**A**). Composition and relative content of VOCs (**B**).

OPLS-DA plots showed a clear separation between the 60% and 80% groups (Figure 4A), with significant differences between the two groups and smaller differences within the 60% group than the 80% group, suggesting that moisture content significantly affects alfalfa silage volatile organic compound composition (R2X = 0.561, R2Y = 0.997, and Q2 = 0.973). The VOCs determined by GC–MS were categorized, and the major VOCs in both groups were terpenes, heterocyclic compounds, alcohols, esters, aldehydes, phenols, ketones, and hydrocarbons (Figure 4B). Among them, terpenes, heterocyclic compounds, alcohols, esters, and phenols were more abundant and were the main odorants in alfalfa silage. The results of the comparison of the contents of the two groups showed that the contents of heterocyclic compounds, phenols, and acids were higher in the 60% group than in the 80% group, and the contents of terpenes, alcohols, and ketones were higher in the 80% group than in the 60% group.

**Figure 5:**
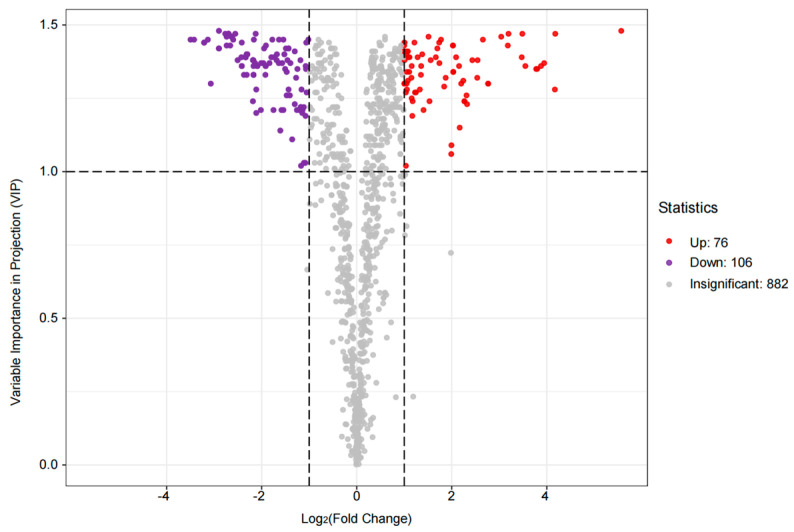
Volcanic map of differential VOCs. The horizontal coordinate represents the logarithmic value of the multiple of the relative content difference of a metabolite in the two groups of samples, and the vertical coordinate represents the VIP value.

The differences in VOC composition between the two groups were significant. Compared to the 80% group, 76 VOCs were up-regulated, 706 VOCs were down-regulated, and 882 VOCs did not change significantly in the 60% group (Figure 5). The different substances with Fold Chang ≥ 2 or ≤0.5, VIP ≥ 1, and *p* < 0.05 were selected for analysis; the up-regulated substances were mainly terpenes, heterocycles, esters, and phenols, and the down-regulated substances were mainly heterocycles, terpenes, alcohols, and ketones (Table S1).

**Figure 6:**
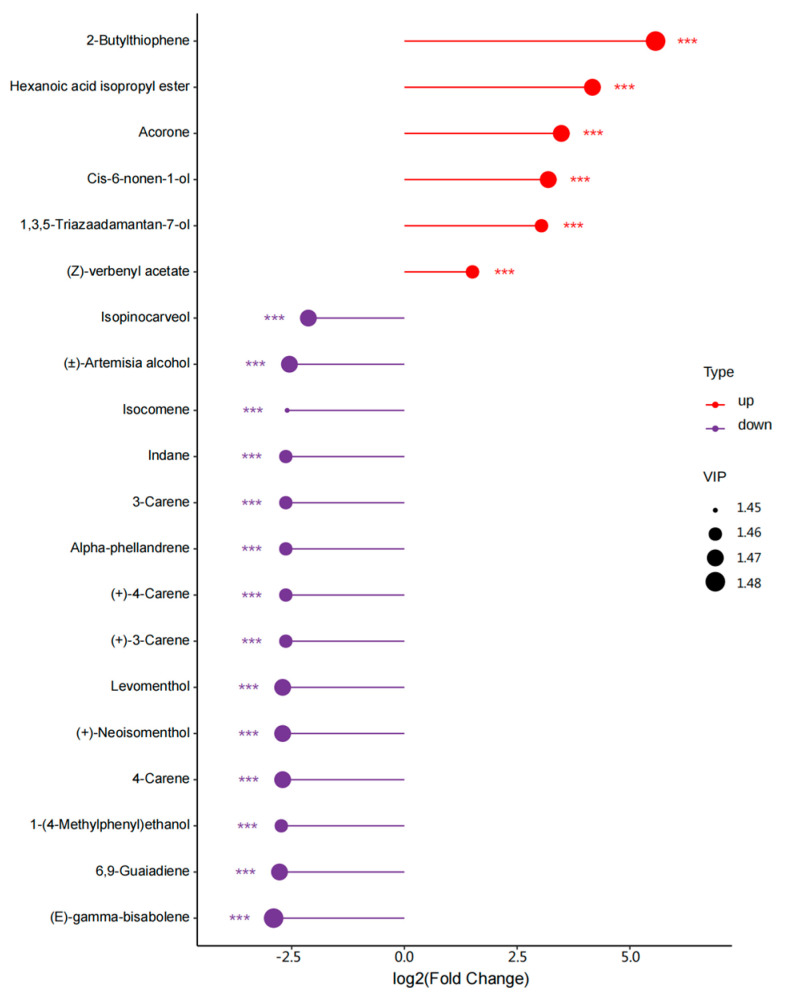
Lollipop map of differential VOCs. The vertical coordinate is the screening substance, the horizontal coordinate is the value of the substance log2FC, the dot size indicates the VIP value of the substance, and *** indicates whether the substance is significant.

The lollipop chart illustrates the top 20 substances with large differences in content (Figure 6). The six substances that were up-regulated included two terpenes (Cis-6-nonen-1-ol and (Z)-verbenyl acetate), two heterocyclic compounds (2-Butylthiophene and Acorone), one ester (Hexanoic acid isopropyl ester), and one alcohol (1,3,5–Triazaadamantan–7-ol). The 14 substances that were down-regulated included nine terpenes (Isopinocarveol, 3-Carene, Alpha-phellandrene, (+)–4–Carene, (+)–3–Carene, Levomenthol, (+)–Neoisomenthol, 4–Carene, and (E)–gamma-bisabolene), three alcohols (Isocomene, 1–(4–Methylphenyl) ethanol, and 6,9–Guaiadiene), one ester ((±)–Artemisia alcohol), and one aromatic compound (Indane).

### 3.6. Functional Annotation and Enrichment Analysis

**Figure 7:**
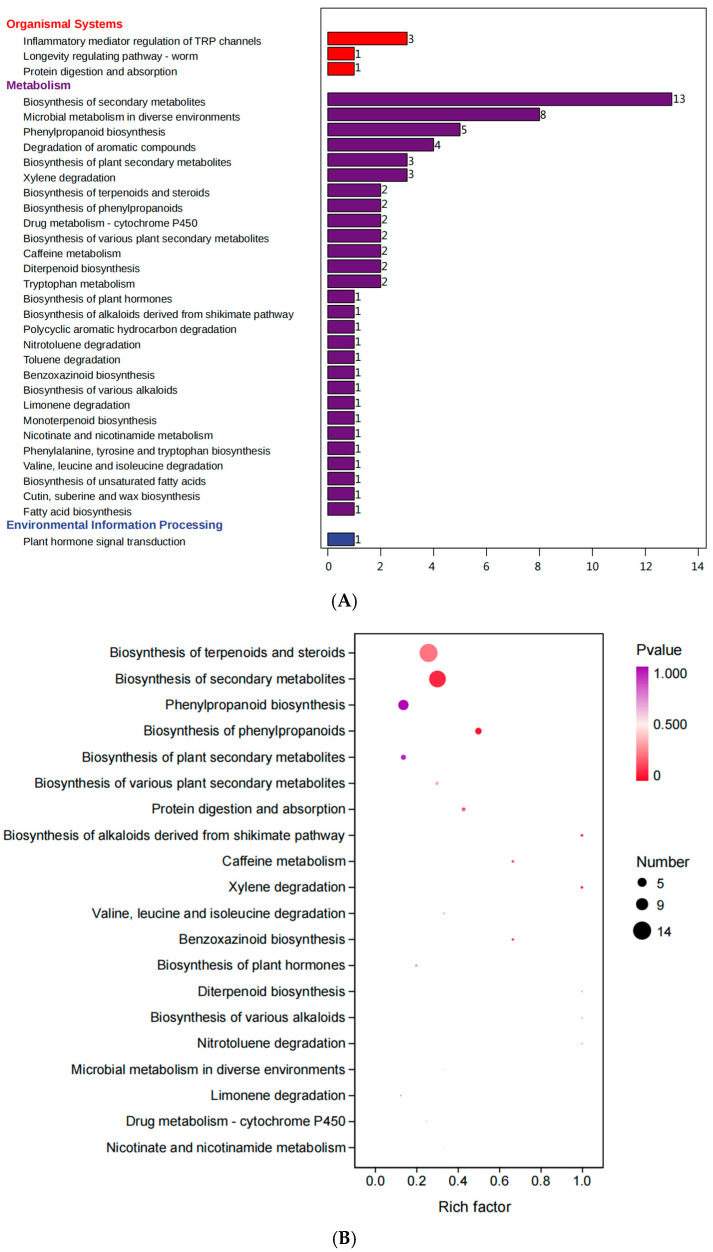
Functional annotation (**A**) and enrichment analysis (**B**) of differential VOCs.

The results of the KEGG annotation of differential VOCs are shown in Figure 7A. The 32 annotated pathways belong to organismal systems, metabolism, and environmental information processing. Among them, the biosynthesis of terpenoids and steroids (13), the biosynthesis of secondary metabolites (8), and phenylpropanoid biosynthesis (5) pathways were more annotated. The KEGG enrichment results of the differential VOCs are shown in Figure 7B. The biosynthesis of terpenoids and steroids, biosynthesis of secondary metabolites, and biosynthesis of phenylpropanoids pathways were enriched for more substances and lower p-values. The phenylpropanoid biosynthesis and biosynthesis of plant secondary metabolite pathways were enriched with more substances.

## 4. Discussion

The results of the odor response intensity of alfalfa silage showed significant differences in odor characteristics between the 60% and 80% moisture content groups. The intensity of response to terpenes, inorganic sulfides, nitrogen oxides, and organic sulfides in alfalfa silage was greater at high moisture content. The fluctuation in nitrogen oxides may be related to the crude protein composition of alfalfa silage. Zhou et al. [23] demonstrated a potential correlation between changes in nitrogen oxides and crude protein breakdown in silage. The process of protein hydrolysis is mainly due to the decomposition of crude proteins facilitated by microorganisms, leading to the production of soluble proteins and the subsequent release of nitrogenous substances. Due to the low dry matter content in 80% moisture content silage, higher oxygen residue, and less effective lactic fermentation, the degradation of proteins by harmful microorganisms cannot be effectively inhibited, resulting in an increase in nitrogen oxides. Urbach [24] observed that the odor of silage ingredients is usually composed mainly of terpenes and sesquiterpenes, which are compounds known for their potent aroma and potential influence on the flavor of silage. The 80% moisture content silage fermentation was less effective in degrading and converting terpenes in the raw material, with silage retaining more grassy and woody odors and less acid and aroma production. High water content may cause anaerobic conditions to worsen, with microorganisms producing more ammonia and sulfide. Faulkner et al. [25] found that terpenes present in animal feed have the ability to influence milk flavor. This effect can occur both as a direct interaction of aroma chemicals and indirectly as precursors of other volatile aromatic compounds, including esters, aldehydes, and alcohols. Therefore, the study of silage odor characteristics and the composition of volatile organic compounds is of great significance for improving the quality and palatability of silage and the flavor of livestock products. The response intensity of inorganic and organic sulfides in 60% moisture content silage fermentation is low, and their effect on silage odor is mainly through the release of hydrogen sulfide, dimethyl sulfide, and other odor substances, which have a pungent odor of rotten eggs, onions, and garlic. Attention needs to be paid to controlling the generation of sulfides during silage in order to reduce the impact on the sensory quality of silage [26]. OPLS-DA results showed that alfalfa silage with different moisture contents could be effectively differentiated by the response of the electronic nose to odor characteristics. Among them, the difference in odor characteristics between 80% moisture content silage and 60% moisture content silage was large, which was consistent with the olfactory sensory results of the researchers, which provided a new idea for silage classification and quality identification.

The results of the silage quality analysis showed that moisture content had a significant effect on silage nutritional quality and fermentation quality. With the increase in moisture content, the dry matter content in silage decreases, which cannot provide sufficient fermentation substrate for lactic acid bacteria, and it is difficult to maintain the appropriate pH value and the balance of microflora, which may trigger the rot and mold of silage. At the same time, low dry matter content means a relative dilution of minerals, vitamins, proteins, and other nutrients in silage, resulting in a decrease in the nutritional value of feed, which will adversely affect the growth, development, and production performance of animals [27]. At 60% moisture content, suitable moisture content provides an ideal fermentation environment for silage, effectively retaining the content of crude protein, soluble sugars, and crude fat. Suitable moisture conditions help to prevent silage from oxygen and weed attacks and can better maintain the stability of nutrients in the feed. In addition, 60% moisture content facilitates the fermentation activity of lactic acid bacteria, which produces lactic acid to lower pH and protect soluble sugars from protein breakdown and fat oxidation [28]. In contrast, alfalfa silage with 80% moisture content has a high fiber content, and fermentation is incomplete due to high humidity, leaving cellulose and other components relatively stable and occupying a large proportion. Fermentation microorganisms, such as lactic acid bacteria, are less active in high moisture environments, making it difficult to effectively decompose soluble carbohydrates, further exacerbating the relative increase in fiber content. Zhao et al. [29] have shown that high-moisture silage is at high risk of nutrient loss and clostridial fermentation, which can lead to increased dry matter loss and protein hydrolysis, as well as increased butyric acid content, reducing feed quality and palatability. Alfalfa silage with an 80% moisture content had a high pH and ammoniacal nitrogen content and low lactic acid and acetic acid content. The high moisture content results in a neutral and alkaline pH in the silage, which is favorable for the release and accumulation of ammoniacal nitrogen, resulting in a high ammoniacal nitrogen content [30]. The production of lactic acid and acetic acid needs to utilize the fermentation effect of probiotics, and incomplete fermentation will lead to the insufficient production of organic acid. Zheng et al. [31] showed that ammonia, butyric acid, and isobutyric acid were higher at high moisture content, whereas lactic acid and acetic acid contents were higher at low moisture content. Therefore, the appropriate moisture content plays a key role in the silage process and can maintain the nutrient content and quality of silage.

The results of bacterial α-diversity showed that Otus, Shannon, Simpson, Chao1, and ACE were higher in the 80% group, indicating that the bacterial species were more diverse and more complex in composition at high moisture content, whereas the bacterial composition was mainly dominated by Lactobacillus at 60% and 70% moisture content. Lactic acid bacteria produce lactic acid during fermentation, which reduces the pH of the environment, making it more acidic and inhibiting most of the stray bacteria, but the lactic acid bacteria themselves are tolerant to acidic environments and can even continue to reproduce at low pH, which makes lactic acid bacteria dominate competitively and become the main bacteria. In contrast, at high moisture content, the pH decreases slowly and the growth of stray bacteria flourishes, leading to a slow fermentation process and poorer silage results, which is in agreement with the findings of Hisham et al. [32].

The results of bacterial composition at the phylum level showed a higher abundance of *Firmicutes* in the 60% group and a higher abundance of *Proteobacteria* in the 80% group. Lactic acid bacteria in the *Firmicutes* were able to rapidly ferment sugars in silage, produce large amounts of lactic acid, and reduce pH, which helped to inhibit the growth of unfavorable microorganisms at an early stage and prevent feed spoilage [33]. In the early stages of silage, *Pseudomonas* and *Enterobacteriaceae* in the *Proteobacteria* will rapidly multiply, competitively consume oxygen and soluble sugars in silage, and slow down the acidification process. At the same time, some bacteria in the *Proteobacteria* may produce amines and other unpleasant volatile substances during metabolism, which may affect the palatability and quality of the feed and even adversely affect animal health [34]. At the genus level, *Pediococcus* and *Lactiplantibacillus* were more abundant in the 60% group, while *Enterococcus* was more abundant in the 70% group. Among them, strains of the genus *Pediococcus* are usually more acid- and stress-tolerant, being able to survive and multiply under low pH and other unfavorable conditions, which makes them perform well in the early and middle fermentation processes of silage. Alhaag et al. [35] found in sweet sorghum and Napier silage that *Pediococcus* was able to reduce ammoniacal nitrogen content and harmful microbial communities effectively and had better silage quality enhancement. *Lactiplantibacillus* was able to rapidly ferment a variety of sugars, decompose complex carbohydrates in the feed, and produce a large amount of lactic acid, thus rapidly lowering the pH value of the feed and improving the nutritional value and digestibility of the feed. *Pediococcus* and *Lactiplantibacillus* could produce not only lactic acid and acetic acid but also other VOCs in the process of silage fermentation [36]. During silage fermentation, *Pediococcus* and *Lactiplantibacillus* produce not only the main lactic and acetic acids, but also other volatile organic acids, ethanol, esters, aldehydes, and ketones, which is in agreement with the findings of Su et al. [37] for corn silage. These compounds work together to give silage a sour, floral, fruity, and other pleasant aroma, enhancing the palatability and quality of the feed. The LEfSe results showed that the differential microorganisms were *Pediococcus pentosaceus* in the 60% group and *Cyanobacteria* in the 80% group. The application of *Pediococcus pentosaceus* helped to enhance the quality and efficiency of silage fermentation by rapidly reducing the pH and inhibiting harmful microorganisms. However, the presence of *Cyanobacteria* interferes with the fermentation process and may also produce toxins that reduce the quality and safety of the feed [38], and under anoxic or anaerobic conditions, denitrifying bacteria are able to reduce nitrates and nitrites to nitrogen while producing N_2_O and NO; these differential microorganisms work together to ultimately affect the quality and odor of the silage.

The bacterial function prediction results showed that at the L2 level, the abundance of protein families regarding genetic information processing, translation, replication and repair, and nucleotide metabolism, was higher in the 60% group. The beneficial microorganisms of alfalfa silage with 60% moisture content generally had higher metabolic activity and, therefore, a greater demand for proteins involved in genetic information processing, translation, replication and repair, and nucleotide metabolism. At the same time, the enzyme activity related to genetic information processing and other metabolic processes may be closer to optimal at this humidity level, with better enzyme function and higher fermentation quality [39]. The abundance of protein families regarding signaling and cellular processes, energy metabolism, and metabolism of cofactors and vitamins was higher in the 80% group, which is due to the fact that the growth of corrupt and less efficient fermenting microorganisms requires robust cellular processes to cope with environmental stress, competition, and energy demands, which requires an increase in proteins involved in energy metabolism to meet these increased demands [40]. An increase in water content affects the diffusion rate of nutrients, changes the way microorganisms acquire and use them, and affects the breakdown of organic matter and cellulose, potentially altering fermentation dynamics and leading to changes in microbial metabolic strategies. At the L3 level, the abundance of transporters, ABC transporters, terpenoid-quinone biosynthesis, porphyrin and chlorophyll metabolism, and platinum drug resistance was higher in the 60% group. This is because the silage fermentation process of the 60% group is faster and the microorganisms may need a more active transport system to effectively absorb nutrients and metabolites from the environment, including more types and a higher abundance of transporters and ABC transporters; the transport system, biosynthetic pathways, and stress response mechanisms may also be enhanced [41]. The peptidases and inhibitors, purine metabolism, ribosome biogenesis, DNA replication proteins, and fatty acid biosynthesis were less abundant in the 60% group. Microbial communities with high water content are more diverse and dynamic, requiring enhanced metabolic activity, including purine metabolism for nucleotide synthesis and fatty acid biosynthesis for membrane construction, with increased demand for DNA replication proteins, ribosome biogenesis, and peptidases involved in protein turnover.

The results of the VOC composition analysis showed that terpenoids, heterocyclic compounds, alcohols, esters, and phenols were the main substances in the two groups. Alfalfa and other legumes are rich in terpenoids, heterocyclic compounds, and phenolic compounds, which are endogenous and secondary metabolites of plants and contribute to the odor composition of alfalfa raw materials. Terpenoids usually have a strong aroma, and depending on their specific structure, they can emit a variety of aroma types, such as floral, citrus, rosin, or herbal aroma [42]. Heterocyclic compounds include pyrazines, thiazoles, etc., which are usually generated during heating or microbial metabolism and may have a nutty, grassy, or smoky smell. A certain degree of non-enzymatic tannin reaction may also occur during silage, which is a chemical reaction between amino acids and reducing sugars that can produce a variety of aromatic compounds with heterocyclic structures [43]. In the silage process, microorganisms such as lactic acid bacteria and yeast ferment basic nutrients such as carbohydrates and proteins in plants to produce a variety of alcohols and esters. Kruis et al. [44] found that lactic acid bacteria and yeast can produce esterification enzymes in the fermentation process, and these enzymes catalyze the esterification reaction between short-chain organic acids and alcohols. Different alcohols have different odors, ethanol has a typical alcoholic odor, higher alcohols may have sweet or fruity odors, and esters often have pleasant fruity and floral odors. These volatile organic compounds combine to form the scent of alfalfa silage. The microbial composition of alfalfa silage at 80% moisture content is more complex; so, stray bacteria use lipids, sugars, and protein metabolism to produce more alcohols and ketones, which, at high concentrations or in the presence of specific compounds, may emit pungent or unpleasant odors that can affect the organoleptic quality of the silage [45].

The results of differential VOC analysis showed that terpenes, heterocycles, esters, and phenols were more abundant in 60% moisture alfalfa silage. Among them, cis-6-nonen-1-ol in terpenes is described as having floral and fruity characteristics, sometimes with citrus and herbal notes. (Z)-verbenyl acetate is commonly found in the essential oils of many plants, especially some herbs and flowers, and the odor is described as having a fresh, floral, and citrus characteristic, often used in perfumes and aromatic products [46]. The heterocyclic compound 2-butylthiophene is commonly used as an ingredient in food flavors and fragrances and has a strong toasted, cereal, and nutty aroma with subtle caramel and sweet notes. Acorone is similar to a mixture of licorice, vanilla, and cinnamon, with a strong and long-lasting fragrance [47]. The hexanoic acid isopropyl ester has a fruity, sweet, and creamy taste and is commonly found in sweets, beverages, and baked goods. These floral, fruity, and sweet volatile organic compounds add aroma and improve sensory quality in silage. Heterocycles, terpenes, alcohols, and ketones are more abundant in the 80% moisture content of alfalfa silage. Among the terpenes, isopinocarveol, 3–carene, and 4–carene are widely found in the essential oils of pine and other pine plants and have piney, camphor, and woody odors. Hammock et al. [48] showed that carene is an important volatile component of basil, with a strong rosin and woody odor. Levomenthol and (+)–neoisomenthol are two common menthol isomers that share many similarities in odor and sensory experience. Both have a cool and minty aroma and are widely used in food, cosmetics, toothpaste, chewing gum, and pharmaceuticals [49]. (E)–gamma-bisabolene is a substance with a pungent odor similar to that of essential oils or resins of plants. Alpha-phellandrene has a black pepper and peppermint aroma and is found in the essential oils of a wide variety of plants, including eucalyptus, pepper, and citrus plants. Among the alcohols, isocomene has a woody odor, 1–(4–methylphenyl) ethanol has a strong alcohol flavor, and 6, 9-guaiadiene has a woody, mossy, and earthy odor [50]. The ester (±)–artemisia alcohol is usually described as having an herbaceous and bitter woody odor, and indane in the aromatic compound has a petroleum irritant odor and sometimes a bitter almond odor [51]. These volatile organic compounds with woody, bitter, and alcoholic odors hinder the formation of aromatic odors during alfalfa silage, thus reducing the sensory quality of the silage, which may account for the poorer odor of silage with high moisture content. By detecting odor response intensity and analyzing VOC composition qualitatively and quantitatively, it was found that high-water-content silage contained more alcohols and ketones, while low-water-content silage contained more esters and phenols. Both electronic nose and GC–MS could distinguish alfalfa silage with different water contents well.

KEGG annotation and enrichment results indicate that differential VOCs are mainly associated with the biosynthesis of terpenoids and steroids, biosynthesis of secondary metabolites, and biosynthesis of phenylpropanoids. Gong et al. [52] determined that terpenes are synthesized via the methylerythritol-phosphate (MEP) and mevalonic acid (MVA) pathways. Specifically, the MEP pathway is responsible for the synthesis of hemiterpenes (C5), monoterpenes (C10), diterpenes (C20), and carotenoid derivatives, while the MVA pathway synthesizes sesquiterpenes (C15), irregular terpenes, and geranyl linalool, and many of the monoterpenes and sesquiterpenes are volatile and are capable of emitting a distinctive aroma. During the silage process, fermentation by microorganisms such as lactic acid bacteria and yeasts transforms and modifies the secondary metabolites of the plant, which affect the final odor profile, and these secondary metabolites mainly include terpenoids, phenolic compounds, alkaloids, and flavonoids. Shang et al. [53] found that in addition to esters, terpenoids and phenols also increased significantly during fermentation and were the main contributors to the floral and sweet flavors. At the same time, the antioxidant properties of secondary metabolites help maintain the quality of silage and prevent oxidative reactions that lead to undesirable odors. Phenylpropanoid compounds are a diverse family of organic compounds derived from the amino acid phenylalanine and play an important role in the aroma profiles of a wide variety of plants and fermentation products. Phenylpropanoids are biosynthesized primarily by the conversion of phenylalanine to cinnamic acid by the enzyme phenylalanine deaminase (PAL), which is then subjected to a variety of modifications to produce different phenylpropane derivatives. Goswami et al. [54] found that the functional metabolic activity of lactic acid bacteria during the fermentation of horse gram sprouts contributes to the production of phenylpropanoid eugenols, which can increase their clove aroma. The difference in water content causes a change in microbial community composition, and microorganisms produce different volatile organic compounds through these metabolic pathways. Therefore, the quality and odor of alfalfa silage can be improved by regulating the composition and metabolism of microorganisms.

## 5. Conclusions

In this study, the effects of different moisture contents on alfalfa silage quality, odor, and bacterial communities were revealed using an electronic nose and GC–MS. The results showed that the 80% group had the worst silage quality and odor and the highest intensity of response to terpenes, sulfides, and nitrogen oxides. The differential VOCs were mainly terpenes, alcohols, and ketones, along with pine, camphor, and menthol odors such as carene and levomenthol. The 60% group had the best silage quality and odor and a significantly increased abundance of *Pediococcus* and *Lactiplantibacillus*. The differential VOCs were mainly heterocyclics, esters, and phenols with fruity, floral, and sweet odors, such as 2-butylthiophene and acorone. The biosynthesis of terpenoids and steroids, biosynthesis of secondary metabolites, and biosynthesis of secondary metabolites are the major metabolic pathways, and appropriate moisture content improves alfalfa silage quality, odor, and microbial community. The electronic nose is suitable for rapid screening and on-site testing, while GC–MS provides in-depth chemical composition analysis, which, in combination, can provide a more comprehensive assessment of silage quality and safety.

## Figures and Tables

**Table 1:** Performance description of the sensor.

Serial Number	Sensor Name	Performance Description
1	W1C	Sensitive to aromatic components
2	W5S	Sensitive to nitrogen oxides
3	W3C	Sensitive to aromatic ammonia
4	W6S	Sensitive to hydrides
5	W5C	Sensitive to aromatic alkanes
6	W1S	Sensitive to methyl groups
7	W1W	Sensitive to terpenes and inorganic sulfides
8	W2S	Sensitive to alcohols, aldehydes, and ketones
9	W2W	Sensitive to organic sulfides
10	W3S	Sensitive to alkanes

**Table 2:** Nutritional quality of alfalfa silage.

Items	50%	60%	70%	80%	SEM	*p*-Value
DM (%FM)	44.57 a	36.41 b	26.8 c	16.53 d	3.164	<0.01
CP (%DM)	17.72 c	18.8 a	18.19 b	17.6 c	0.145	<0.01
NDF (%DM)	34.94 b	32.54 d	33.52 c	35.12 a	0.321	<0.01
ADF (%DM)	27.64 b	26.1 c	26.28 c	28.05 a	0.254	<0.01
WSC (%DM)	2.5 b	2.96 a	2.9 a	2.34 c	0.08	<0.01
EE (%DM)	1.25 b	1.65 a	1.6 a	1.2 b	0.055	<0.01

Fresh matter content (FM), Dry matter content (DM), crude protein (CP), acid detergent fiber (ADF), neutral detergent fiber (NDF), water soluble carbohydrates (WSC), and crude fat (EE). SEM: standard error of the mean. Different letters indicate significant differences between treatments.

**Table 3:** Fermentation quality of alfalfa silage.

Items	50%	60%	70%	80%	SEM	*p*-Value
pH	4.81 b	4.6 c	4.64 c	4.94 a	0.041	<0.01
LA (g/kg DM)	14.88 b	17.4 a	17.27 a	14.31 c	0.419	<0.01
AA (g/kg DM)	3.35 c	4.15 a	3.96 b	3.22 d	0.12	<0.01
NH_3_–N (g/kg TN)	0.85 b	0.63 c	0.68 c	0.96 a	0.04	<0.01

Dry matter content (DM), Total nitrogen (TN), Lactic acid (LA), acetic acid (AA), and ammonia nitrogen (NH3-N). Propionic acid and butyric acid concentrations were not detected in the experiment. SEM: standard error of the mean. Different letters indicate significant differences between treatments.

**Table 4:** α-Diversity of alfalfa silage.

Items	50%	60%	70%	80%	SEM	*p*-Value
Otus	204 c	231 b	213 c	260 a	7.204	<0.01
Shannon	3.417 a	3.223 b	2.774 c	3.447 a	0.106	<0.01
Simpson	0.753 a	0.756 a	0.609 b	0.765 a	0.024	<0.01
Chao1	242.657 d	268.101 b	258.69 c	308.652 a	8.027	<0.01
ACE	251.964 d	282.394 b	266.388 c	318.264 a	8.373	<0.01
Coverage	0.99	0.99	0.99	0.99	<0.01	–

Different letters indicate significant differences between treatments.

## Data Availability

The datasets generated during the current study are available in the [NCBI] repository [https://www.ncbi.nlm.nih.gov/, accessed on 3 January 2025, PRJNA1121500].

## Supplementary Materials

The following supporting information can be downloaded at: https://www.mdpi.com/article/10.3390/microorganisms13020381/s1, Table S1: 181 differential volatile organic compounds.
